# Biophysical and biochemical properties of Deup1 self-assemblies: a potential driver for deuterosome formation during multiciliogenesis

**DOI:** 10.1242/bio.056432

**Published:** 2021-03-03

**Authors:** Shohei Yamamoto, Ryoichi Yabuki, Daiju Kitagawa

**Affiliations:** Department of Physiological Chemistry, Graduate School of Pharmaceutical Sciences, The University of Tokyo, Hongo, Tokyo 113-0033, Japan

**Keywords:** Biomolecular condensates, Centriole, Deuterosome, Multiciliogenesis, Self-assembly

## Abstract

The deuterosome is a non-membranous organelle involved in large-scale centriole amplification during multiciliogenesis. Deuterosomes are specifically assembled during the process of multiciliogenesis. However, the molecular mechanisms underlying deuterosome formation are poorly understood. In this study, we investigated the molecular properties of deuterosome protein 1 (Deup1), an essential protein involved in deuterosome assembly. We found that Deup1 has the ability to self-assemble into macromolecular condensates both *in vitro* and in cells. The Deup1-containing structures formed in multiciliogenesis and the Deup1 condensates self-assembled *in vitro* showed low turnover of Deup1, suggesting that Deup1 forms highly stable structures. Our biochemical analyses revealed that an increase of the concentration of Deup1 and a crowded molecular environment both facilitate Deup1 self-assembly. The self-assembly of Deup1 relies on its N-terminal region, which contains multiple coiled coil domains. Using an optogenetic approach, we demonstrated that self-assembly and the C-terminal half of Deup1 were sufficient to spatially compartmentalize centrosomal protein 152 (Cep152) and polo like kinase 4 (Plk4), master components for centriole biogenesis, in the cytoplasm. Collectively, the present data suggest that Deup1 forms the structural core of the deuterosome through self-assembly into stable macromolecular condensates.

This article has an associated First Person interview with the first author of the paper.

## Abstract (precis)

**Summary:** Deuterosome protein 1 (Deup1) self-assembles into stable condensates that can be the structural core of the deuterosome during multiciliogenesis.

## INTRODUCTION

Multiciliated cells are found in numerous animals and in various organs, such as the brain, trachea, and oviduct (Afzelius, 1976). The directional movement of multiple cilia generates extracellular fluid flow for various physiological processes, including mucus removal and transportation of oocytes (Afzelius, 1976; Dirksen and Satir, 1972; Reiter and Leroux, 2017). In multiciliogenesis, the number of centrioles that act as basal bodies for cilia formation are amplified to generate hundreds of cilia through multiple mechanisms, including pre-existing centriole-dependent and deuterosome-dependent pathways (Boutin and Kodjabachian, 2019; Klos Dehring et al., 2013; Mercey et al., 2019a; Nanjundappa et al., 2019; Zhao et al., 2013).

The deuterosome is a non-membranous structure that is specifically formed during multiciliogenesis (Spassky and Meunier, 2017). Electron microscopy studies have determined that deuterosomes are electron-dense granular structures that associate with multiple centrioles and are several hundred nanometers in diameter (Funk et al., 2015; Anderson and Brenner, 1971; Dirksen, 1971; Kalnins and Porter, 1969; Sorokin, 1968; Steinman, 1968). It is thought that *de novo* centriole biogenesis occurs around deuterosomes to support the large-scale centriole biogenesis that occurs in multiciliogenesis (Klos Dehring et al., 2013; Zhao et al., 2013). On the other hand, paradoxically, a recent study revealed that the large-scale centriole biogenesis can occur even in the absence of deuterosomes (Mercey et al., 2019a). Recent studies have also shown that deuterosomes are assembled even in the absence of pre-existing centrioles (Mercey et al., 2019b; Nanjundappa et al., 2019; Zhao et al., 2019). However, the biochemical nature of deuterosomes and the molecular mechanisms of deuterosome formation are poorly understood.

Deuterosome protein 1 (Deup1) has been identified as a component of the deuterosome (Zhao et al., 2013). Deup1 is specifically expressed during multiciliogenesis and is essential for deuterosome formation (Mercey et al., 2019a; Zhao et al., 2013). Thus far, Deup1 has been recognized as a sole and specific molecular marker for deuterosomes (Zhao et al., 2013, 2019). The C-terminal region of Deup1 binds to centrosomal protein 152 (Cep152), a critical protein for centriole biogenesis (Blachon et al., 2008; Cizmecioglu et al., 2010; Klos Dehring et al., 2013; Zhao et al., 2013). Nevertheless, the mechanism through which Deup1 contributes to deuterosome assembly remains unclear.

Condensation of protein and/or nucleic acids is a key process for the formation of non-membranous organelles (Banani et al., 2017; Woodruff et al., 2018). Biomolecular condensates exhibit various material properties, such as liquid-like, gel-like, and solid states (Boeynaems et al., 2018; Woodruff et al., 2018). It has been reported that non-dynamic stable condensates (e.g. centrosomes and nuclear pores) act as scaffolds that selectively compartmentalize specific molecules and regulate specific biochemical reactions (Boke et al., 2016; Laos et al., 2015; Schmidt and Görlich, 2015; Woodruff et al., 2017, 2018). Although Deup1 forms macromolecular structures when ectopically expressed in bacteria cells (Zhao et al., 2013), little is known about the self-assembly of Deup1, and the relationship between biomolecular condensation and deuterosome formation remains unknown.

This study investigated the molecular properties of Deup1, and found that the material properties of Deup1-positive assemblies are in a non-dynamic state in multiciliogenesis. Our cell-biological and biochemical analyses demonstrated that Deup1 has the ability to self-assemble into non-dynamic condensates both *in vitro* and in cells. We also showed that the Deup1 N-terminus confers its condensation properties. We demonstrated that self-assembly of Deup1 drives the compartmentalization of Cep152 and polo like kinase 4 (Plk4), master proteins for centriole biogenesis, in the cytoplasm. Collectively, the present data propose that Deup1 self-assembly acts as a structural core for deuterosome formation in multiciliogenesis.

## RESULTS

### Deup1 forms stable assemblies during multiciliogenesis

**Fig. 1.**
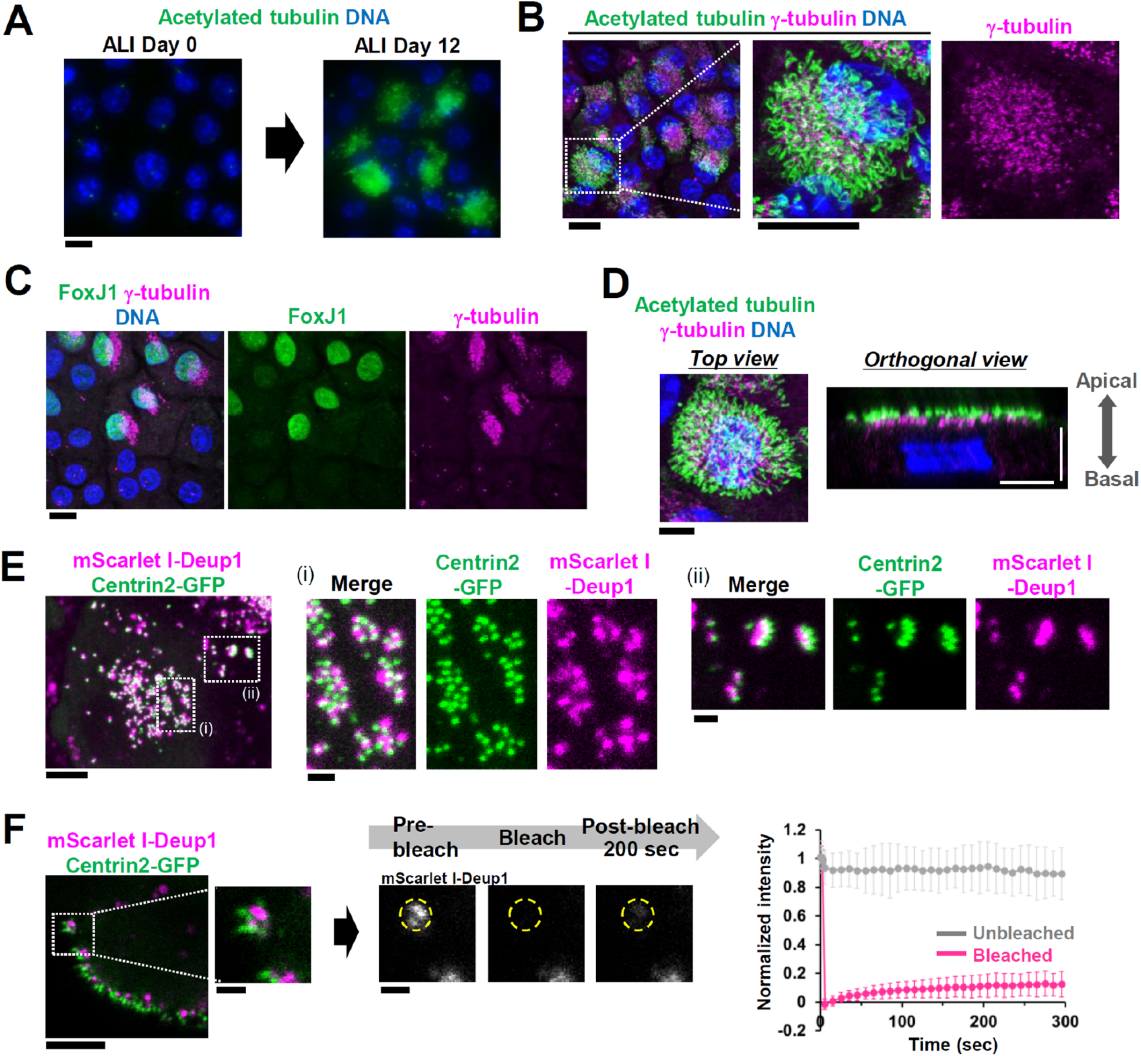
**Deup1 forms stable assemblies during multiciliogenesis.** (A) Differentiation of E1 cells. E1 cells were fixed and immunostained before (ALI 0 days) and after (ALI 12 days) differentiation. Scale bar, 10 µm. Green and blue represent acetylated tubulin and DNA, respectively. (B) Representative image of differentiated E1 cells. E1 cells were cultured in ALI for 21 days and fixed for immunostaining. Scale bar, 10 µm. In merged images, green, magenta and blue represent acetylated tubulin, γ-tubulin and DNA, respectively. (C) Immunostaining of FoxJ1 in differentiated E1 cells cultured in ALI for 21 days. Scale bar, 10 µm. In merged images, green, magenta and blue represent FoxJ1, γ-tubulin and DNA, respectively. (D) Representative image of the top view (left) and the orthogonal view (right) of differentiated E1 cells (ALI 21 days). Positions of basal bodies and cilia are polarized along apico-basal axis. Scale bar, 5 µm. (E) Live imaging of differentiating E1 cells expressing mScarlet I-Deup1 and Centrin2-GFP in (ALI 5 days). Scale bar, 5 µm; magnified image, 1 µm. (F) FRAP analysis of mScarlet I-Deup1 in differentiating E1 cells (ALI 5 days). Cells showing association of centrin foci with Deup1 foci were analyzed. Scale bar, 5 µm; magnified image, 1 µm. Intensities were normalized with the average of three pre-bleach signals. Graph shows mean±s.d. of 50 regions from 31 cells (from three independent experiments).

We used the E1 cell line, a clonal cell line derived from oviductal epithelium of a *p53* knockout mouse, to perform cell-biological analyses of multiciliogenesis (Nakano et al., 2017; Umezu et al., 2010). A previous study showed that E1 cells have the capacity to differentiate into ciliated cells in air-liquid interface (ALI) cultures (Nakano et al., 2017). We first confirmed that E1 cells differentiated into multiciliated cells in ALI culture, and could observe both centriole amplification and cilia formation (Fig. 1A,B; Fig. S1A). The cells in which centriole amplification was observed were forkhead box J1 (FoxJ1)-positive, suggesting that E1 cells differentiated into multiciliated cells through the canonical FoxJ1-mediated pathway (Fig. 1C). Of note, FoxJ1 is an essential transcription factor for multiciliogenesis (Yu et al., 2008). In addition, cilia and centrioles were positioned at the apical side of cells, suggesting that apico–basal polarity was established in differentiated E1 cells (Fig. 1D). These findings were similar to those observed in oviductal epithelium (Dirksen and Satir, 1972). Multiple Deup1 foci were associated with centrin foci in the differentiation of E1 cells, as observed in other primary culture cells (Fig. S1D, E) (Zhao et al., 2013, 2019). From these results, we decided to use the E1 cell line as a model system for the study of multiciliogenesis.

We analyzed the dynamics of Deup1-positive assemblies (recognized as deuterosomes) to investigate their biophysical properties. Through lenti-virus-mediated gene transfer, we generated E1 cells that expressed Deup1 and Centrin2 tagged with mScarlet I and GFP, respectively, with a doxycycline-inducible expression promoter (Fig. S1F,G). Using live cell imaging, we clearly observed mScarlet I-Deup1 foci associated with Centrin2-GFP foci in differentiating E1 cells (ALI for 5 days) (Fig. 1E). With this cell line, we performed fluorescence recovery after photobleaching (FRAP) analysis of mScarlet I-Deup1 in differentiating cells (ALI for 5 days) that had multiple centrin foci. We found that the turnover of mScarlet I-Deup1 was quite low, even 290 s after photo-bleaching [recovery: 12±9% (mean±standard deviation)] (Fig. 1F; Fig. S2), suggesting that Deup1 assemblies are highly stable structures.

**Fig. 2.**
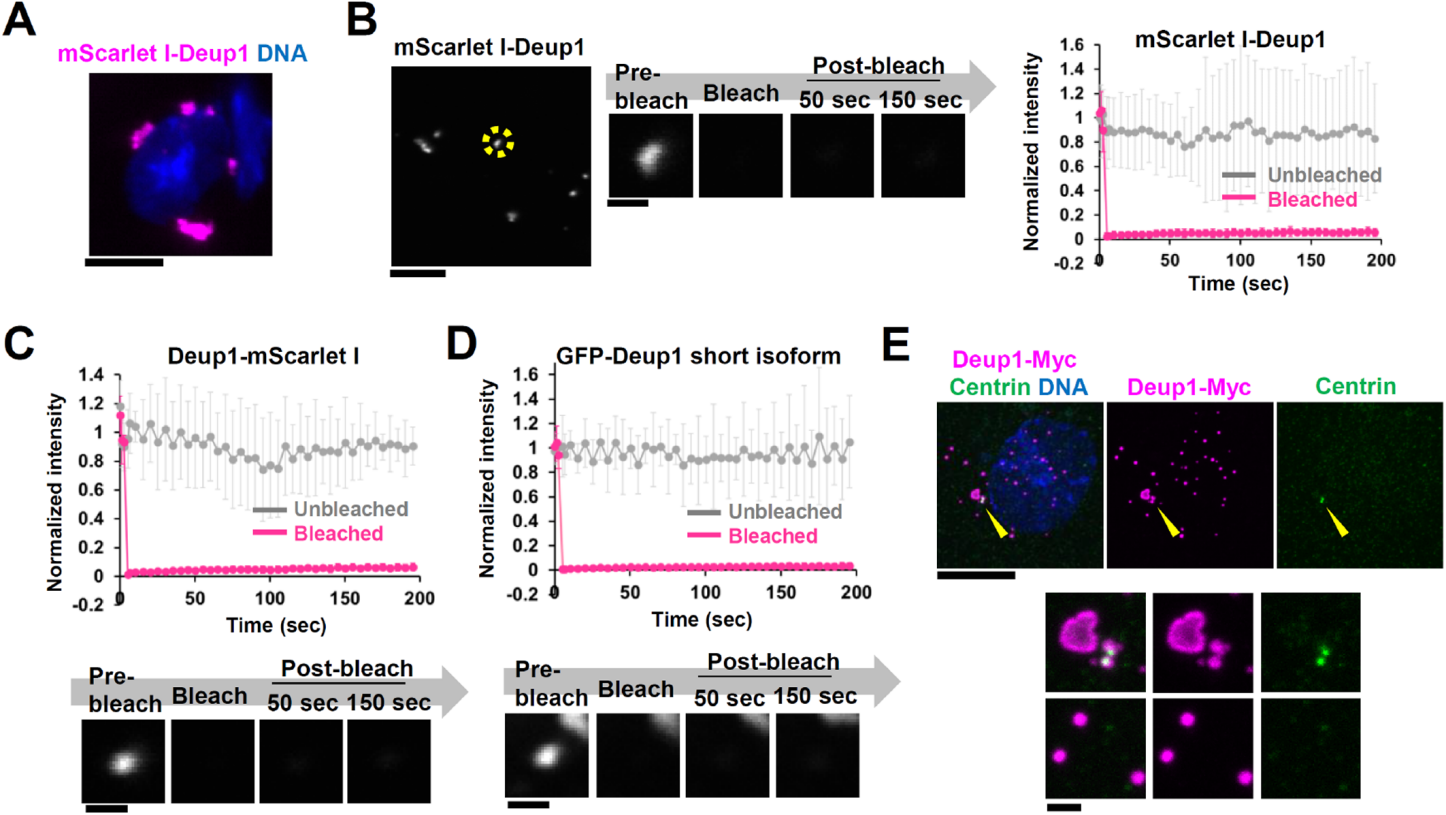
**Ectopically expressed Deup1 forms non-dynamic assemblies in undifferenciated cells.** (A) Ectopic expression of mScarlet I-Deup1 in undifferentiated E1 cells. Scale bar, 10 µm. (B)-(D) FRAP analysis of (B) mScarlet I-Deup1, (C) Deup1-mScarlet I and (D) GFP-Deup1 short isoform in undifferentiated E1 cells. Scale bar, 5 µm; magnified image, 1 µm. Intensities were normalized with the average of three pre-bleach signals. Graphs show mean±s.d. of (B) 16 cells and (C)-(D) 8 cells from two independent experiments. (E) Undifferentiated E1 cells expressing Deup1-Myc. Cells were stained with anti-Myc and anti-Centrin antibodies. Scale bar, 10 µm; magnified image, 1 µm.

It has been previously reported that Deup1 assembles into macromolecular structures even in undifferentiating cells ectopically expressing Deup1 (Zhao et al., 2013). We confirmed that ectopically-expressed Deup1 formed macromolecular structures in undifferentiated E1 cells (Fig. 2A; Fig. S1B, C). We performed FRAP analysis of mScarlet I-Deup1 in undifferentiated E1 cells to further characterize the properties of Deup1 assemblies. We found that, even in the undifferentiated cells, mScarlet I-Deup1 showed a slow turnover within the Deup1 assemblies (recovery: 6±3% at 290 s after photobleaching) (Fig. 2B). This result indicates that Deup1 has the ability to form stable assemblies even in the cytoplasm of undifferentiated cells. Although there is a possibility that fluorescence protein tagging or an alternate isoform of Deup1 may influence the dynamics of Deup1, we confirmed that Deup1 formed static assemblies in the cytoplasm even with C-terminus tagging (Deup1-mScarlet I), GFP tagging, and a shorter isoform of Deup1 (Fig. 2C,D; Fig. S3A, B). Importantly, some Deup1 assemblies were not associated with centrin foci in undifferentiated cells (Fig. 2E). This result implies that the Deup1 assemblies are formed independently of the pre-existing centrioles, which is consistent with recent findings showing that the deuterosomes themselves form independently of the presence of pre-existing centrioles (Mercey et al., 2019b; Nanjundappa et al., 2019; Zhao et al., 2019).

### Deup1 self-assembles into non-dynamic condensates *in vitro*

**Fig. 3.**
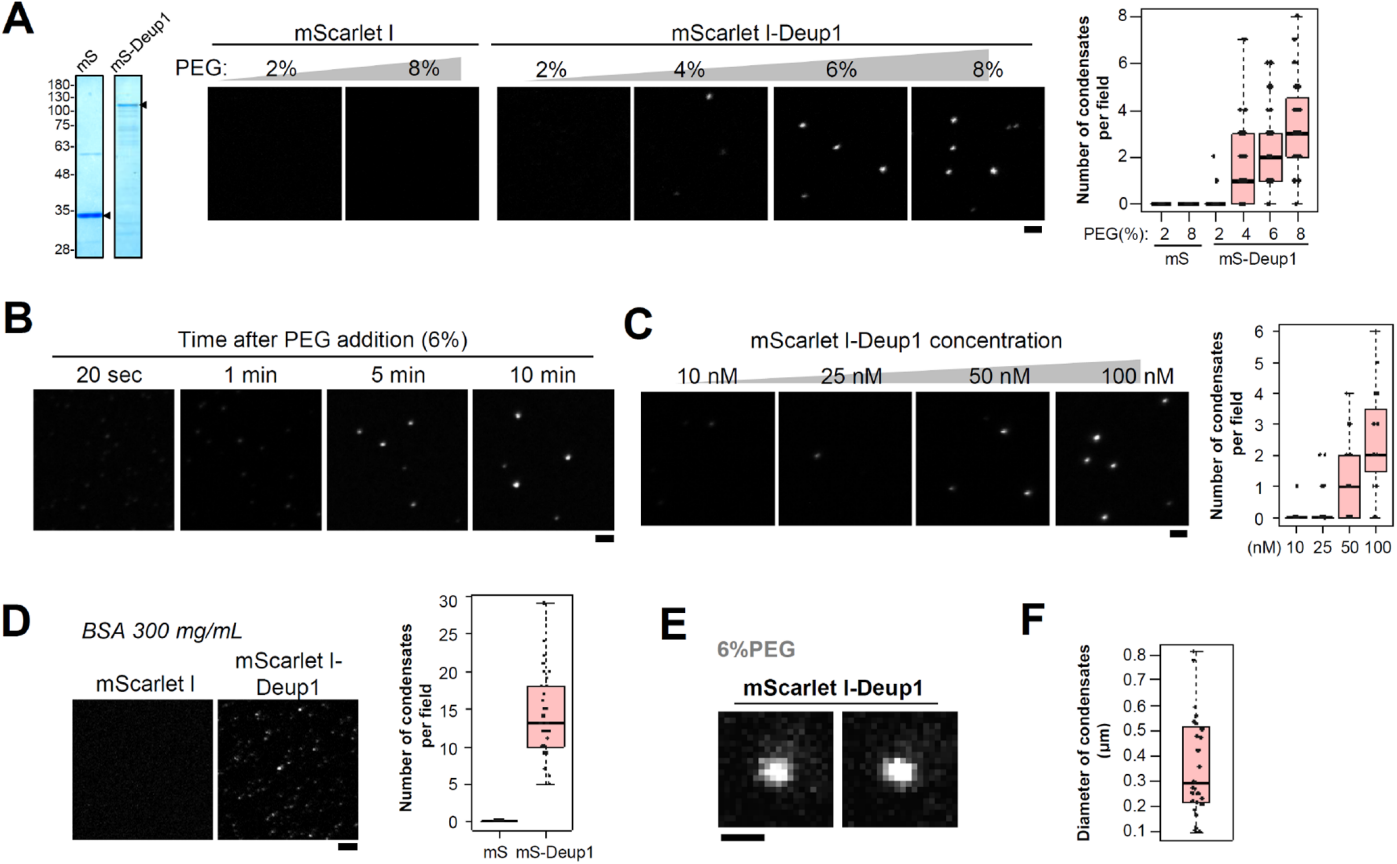
**Deup1 self-assembles into condensates *in vitro*.** (A) Purified mScarlet I-Deup1 protein forms macro-molecular structures in the presence of PEG. Left, CBB staining of purified proteins. Arrowheads refer to the purified target proteins. Right, Representative images of 100 nM purified mScarlet I (mS) and mScarlet I-Deup1 (mS-Deup1). The indicated concentration of PEG was added into the buffer solution. *n*=40, 36, 40, 40, 32 and 40 fields. Scale bar, 2 µm. (B) 100 nM of purified mScarlet I-Deup1 protein was incubated in a buffer solution containing 6% PEG. Time after PEG addition. Scale bar, 2 µm. (C) Effects of the concentration of Deup1 in the presence of 6% PEG. *n*=28, 32, 32 and 32 fields. Scale bar, 2 µm. (D) Effects of BSA (300 mg/ml) on mScarlet I-Deup1 *in vitro*. Scale bar, 2 µm. *n*=32 fields per condition. (E) Magnified images of mScarlet I-Deup1 assemblies formed in the presence of 6% PEG. Scale bar, 0.5 µm. (F) Quantification of the diameter of Deup1 assemblies formed *in vitro* (6% PEG). *n*=27 condensates. Graphs show box (25 to 75%), whisker (10 to 90%). Lines in graphs indicate medians.

Deup1 assemblies can be formed by ectopic expression of Deup1 in undifferentiated cells. Thus, we hypothesized that Deup1 itself forms macromolecular assemblies through self-assembly. To address this, we analyzed the biochemical properties of the purified mScarlet I-Deup1 protein (Fig. 3A and Fig. S4A). We used polyethylene glycol (PEG) as a molecular crowding reagent to investigate the properties of Deup1 in an environment with molecular crowding that mimics the cytoplasmic state. We found that, in the presence of PEG, Deup1 assembled into macromolecular structures *in vitro*, whereas mScarlet I alone did not form such structures (Fig. 3A,B). The frequency of formation of Deup1 assemblies depended on the concentrations of PEG and Deup1 (Fig. 3A and C). In addition, a high concentration of bovine serum albumin (BSA), which also functions as a molecular crowding reagent, promoted Deup1 self-assembly (Fig. 3D) (Nishizawa et al., 2017; Wieczorek et al., 2013). These Deup1 assemblies were several hundreds of nanometers in diameter (Fig. 3E,F). These results suggest that Deup1 possesses the property to self-assemble into macro-molecular structures in molecular crowding conditions.

**Fig. 4.**
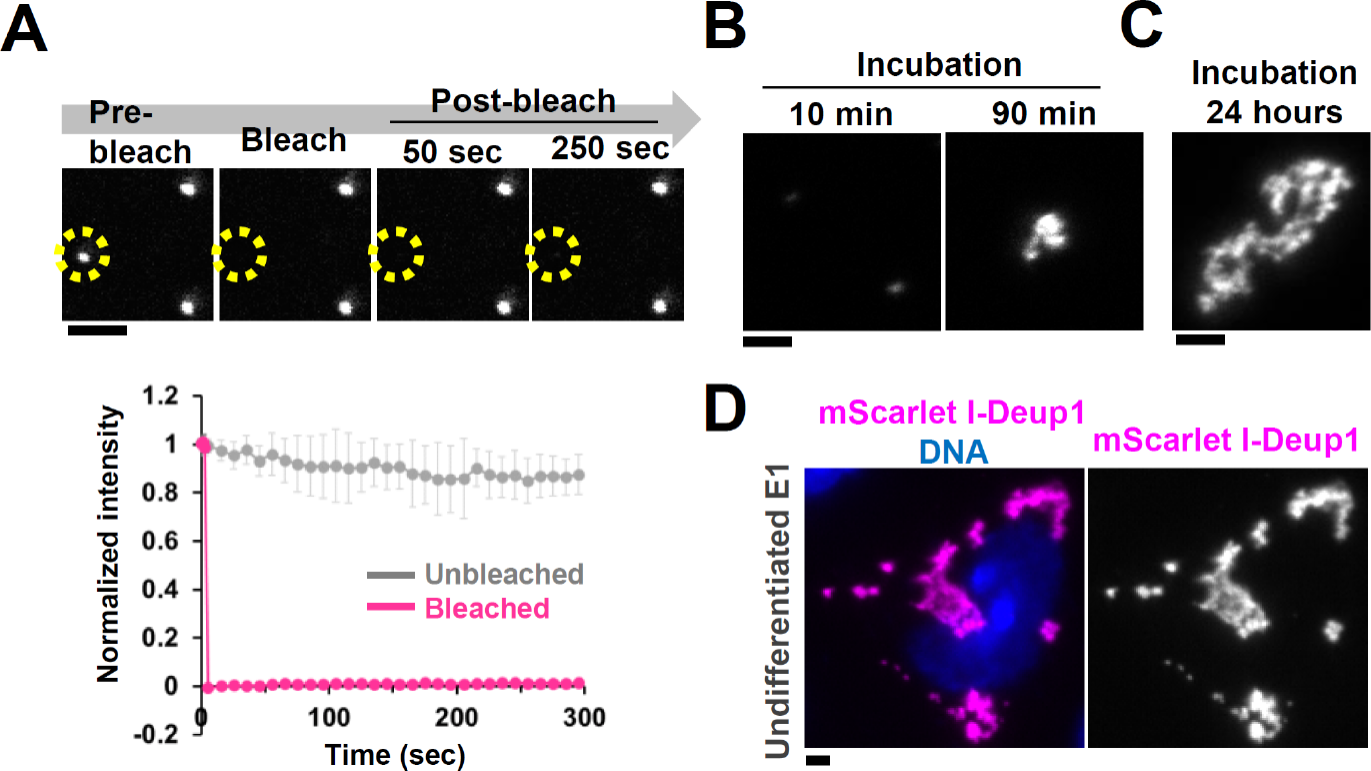
**Deup1 forms non-dynamic condensates *in vitro*.** (A) FRAP analysis of mScarlet I-Deup1 formed in the presence of 6% PEG. Intensities were normalized with the average of three pre-bleach signals. Graph shows mean±s.d. of 9 condensates from two independent experiments. Scale bar, 2 µm. (B) and (C) Effects of incubation of mScarlet I-Deup1. mScarlet I-Deup1 was incubated for the indicated time in the presence of 6% PEG. Scale bar, 2 µm. (D) Representative image of mScarlet I-Deup1 ectopically expressed in undifferentiated cells. Scale bar, 2 µm.

We performed FRAP analysis of mScarlet I-Deup1 to investigate the biophysical properties of the Deup1 assemblies formed *in vitro*. Consistent with the results observed in cells, we found that turnover of mScarlet I-Deup1 was barely observed within the Deup1 assemblies *in vitro* (recovery: 1±1% t 290 s after photobleaching) (Fig. 4A). In addition, internal rearrangement in Deup1 assemblies was undetectable following partial bleaching of the fluorescence of mScarlet I-Deup1 assemblies (Fig. S4B). We also found that, after longer-term incubations with Deup1 (90 min and 24 h), Deup1 foci were subsequently assembled into larger and more irregular structures (Fig. 4B,C). It has been shown that liquid-like biomolecular condensates are assembled into spherical droplet-like structures (Boeynaems et al., 2018; Woodruff et al., 2018). Therefore, our results suggest that Deup1 assemblies are not liquid-like droplets but, rather, are gel-like or solid structures. Similar irregular structures of Deup1 were observed in undifferentiated E1 cells expressing mScarlet I-Deup1, suggesting that the Deup1 assemblies formed *in vitro* share similar material properties with Deup1 condensates in cells (Fig. 4D). We confirmed that a shorter isoform of Deup1 also self-assembled into static structures *in vitro* (Fig. S4C,D). These results suggest that Deup1 possesses the ability to self-assemble into non-dynamic condensates both *in vitro* and in cells.

We also tested whether the Deup1 assemblies formed *in vitro* functioned as a scaffold for the recruitment of a critical centriole component. For this purpose, we examined whether the *in vitro* self-assembled Deup1 has the capacity to recruit Cep152 (a known interactor) into the structure (Zhao et al., 2013). As expected, Cep152-SNAP-Halo was selectively concentrated on the self-assembled Deup1 assemblies compared with SNAP-Halo alone (Fig. S4E). These results suggest that Deup1 self-assembly is sufficient for concentrating Cep152 *in vitro* and in cells.

### The N-terminus of Deup1 confers its self-assembly property

**Fig. 5.**
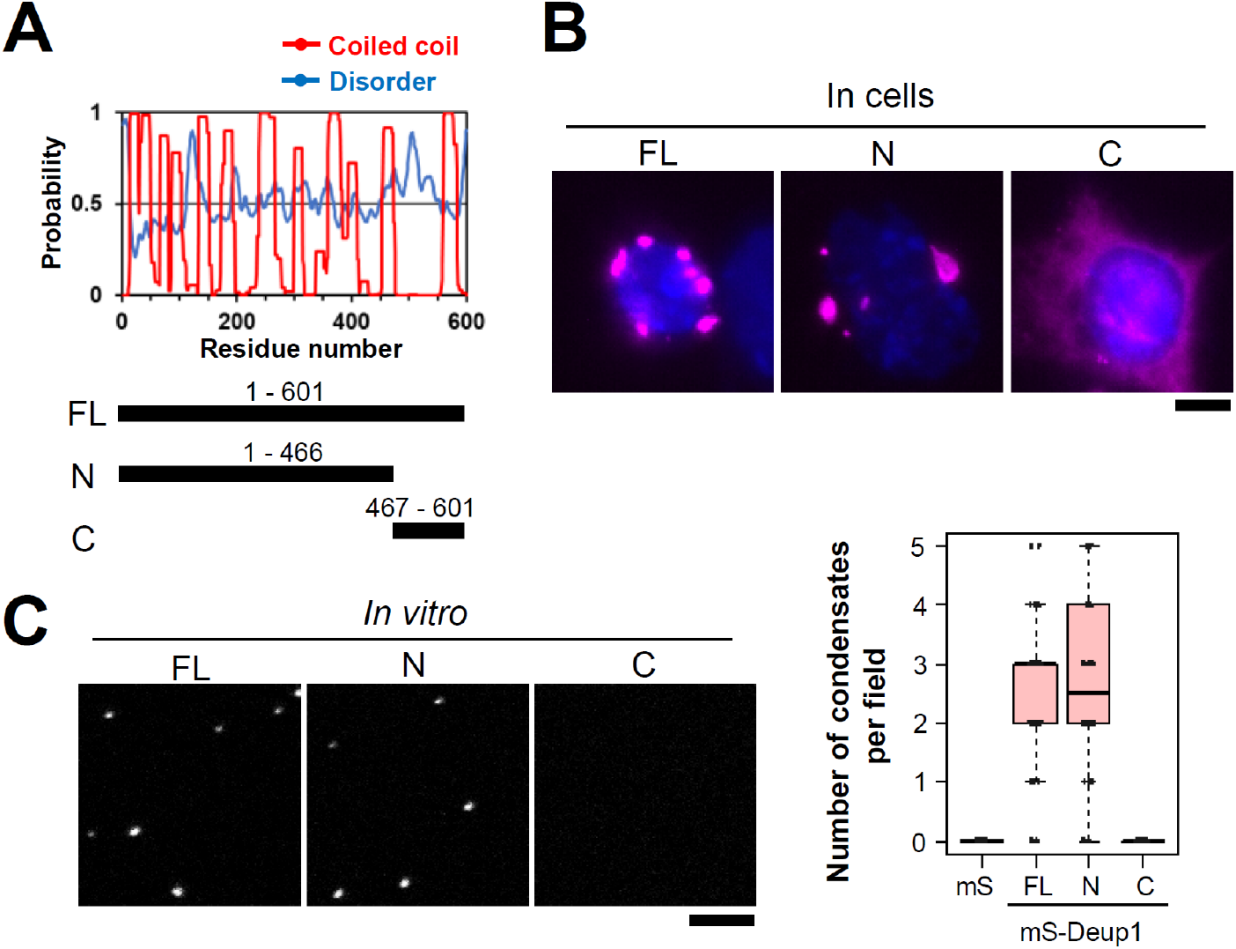
**Deup1 self-assembles via its N-terminus.** (A) Prediction of coiled coil and disordered regions in mouse Deup1 protein. Regions of Deup1 fragments are shown. N-terminus, 1-466 a.a.. C-terminus, 467-601 a.a.. (B) mScarlet I-Deup1 fragments were ectopically expressed in undifferentiated E1 cells. Magenta and blue represent mScarlet I-Deup1 and DNA, respectively. Scale bar, 5 µm. (C) Condensation of purified mScarlet I-Deup1 fragments in the presence of 6% PEG. Graphs show box (25 to 75%), whisker (10 to 90%). Lines in graphs indicate medians. *n*=40 fields per condition. Scale bar, 5 µm.

We constructed several fragments of Deup1 and ectopically expressed mScarlet I-Deup1 fragments in undifferentiated E1 cells to identify the domains of Deup1 that confer the ability to self-assemble (Fig. S5A– C). Unexpectedly, most Deup1 fragments we designed assembled into macromolecular structures in the cytoplasm (Fig. S5A– C). These results suggest that Deup1 assembles through multiple interactions between Deup1 molecules. Our comprehensive analysis revealed that the C-terminus fragment (467–601 a.a.) of Deup1, containing the reported Cep152 binding region, seldom assembled into macromolecular structures alone and, rather, showed a diffuse distribution in undifferentiated E1 cells (Fig. 5A,B). In contrast, the N-terminus fragment (1–466 a.a.) of Deup1, containing multiple coiled coil domains, showed self-assembly properties similar to those of full-length Deup1 (Fig. 5A,B). The purified N-terminus fragment of Deup1 also showed strong self-assembly properties *in vitro* (as observed with full-length Deup1), whereas the C-terminus fragments did not (Fig. 5C; Figs S4C and S5E). Moreover, ectopically expressed human Deup1 also assembled into macromolecular structures via its N-terminus in HeLa cells, suggesting that human Deup1 also possesses similar properties to those of mouse Deup1 (Fig. S5F, G). These results suggest that Deup1 self-assembles via its N-terminus region, presumably through multiple interactions between Deup1 molecules.

### Self-assembly of Deup1 via its N-terminus can drive spatial compartmentalization of Cep152 and Plk4 in the cytoplasm

**Fig. 6.**
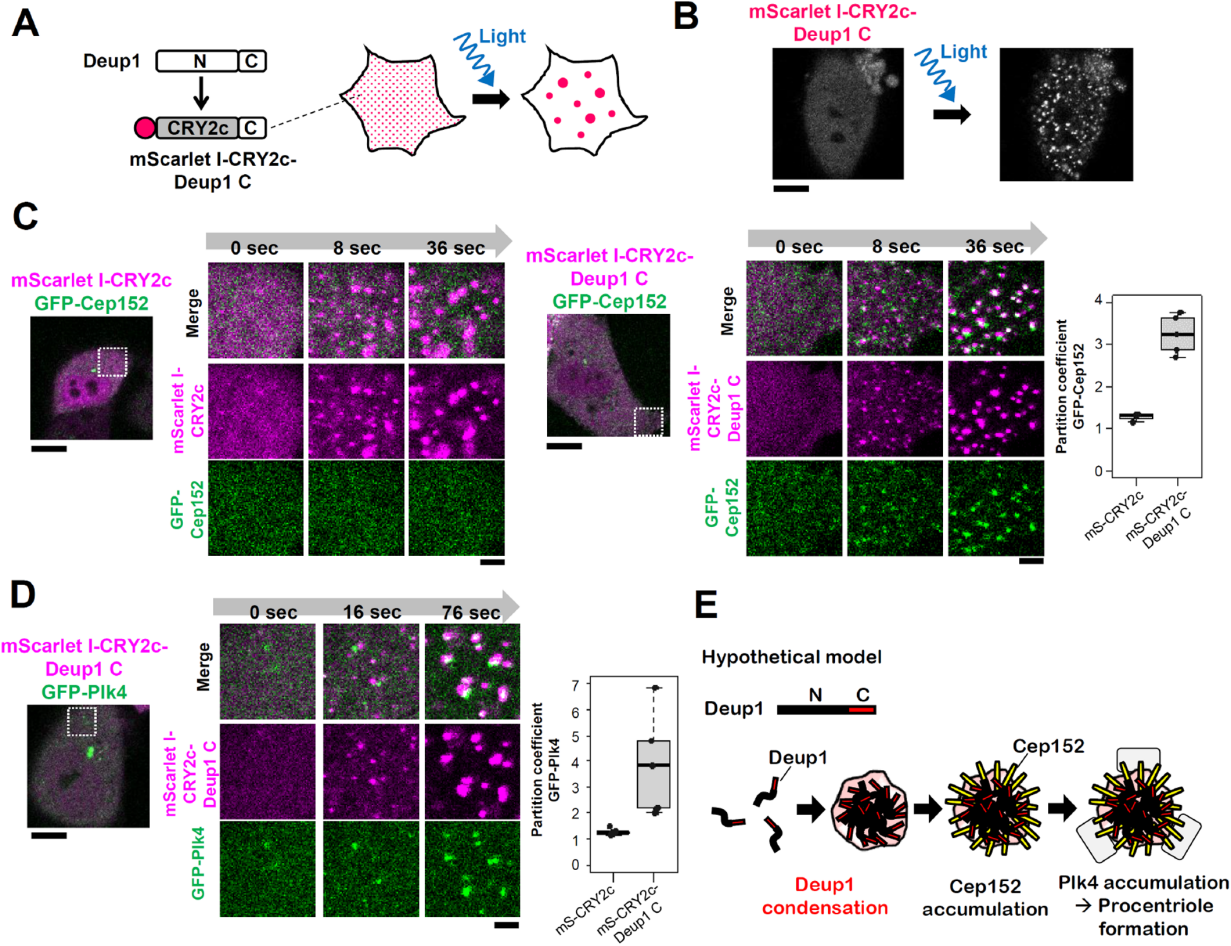
**Deup1 self-assembly via its N-terminus induces accumulation of Cep152 and Plk4 in the cytoplasm.** (A) Schematic of the experimental design. Human Deup1 N-terminus region (1-468 a.a.) was replaced with CRY2clust (CRY2c). (B) Representative image of blue light induced condensation of mScarlet I-CRY2clust-Deup1-C (469-604 a.a.) was shown. Scale bar, 10 µm. (C) Co-expression of GFP-Cep152 with mScarlet I-CRY2clust or mScarlet I-CRY2clust-Deup1-C in HeLa cells. Graph indicates partition coefficient of GFP-Cep152 in CRY2clust condensates at 36 s. mScarlet I-CRY2clust, *n*=4 cells. mScarlet I-CRY2clust-Deup1 C, *n*=5 cells from two independent experiments. (D) Co-expression of GFP-Plk4 with mScarlet I-CRY2clust-Deup1 C in HeLa cells. Cep152-SNAP was co-expressed in cells. Graph indicates partition coefficient of GFP-Plk4 in CRY2clust condensates at 76 s. *n*=5 cells from two independent experiments per condition. (C)-(D) Time after the induction with blue light is shown. Non-magnified images show 0 s after blue light. Scale bar, 10 µm; magnified image, 2 µm. Graphs show box (25 to 75%), whisker (10 to 90%). Lines in graphs indicate medians. (E) Hypothetical model. In multiciliogenesis, Deup1 expression induces Deup1 self-assembly via its N-terminus in the cytoplasm. The Deup1 assemblies act as scaffolds for procentriole assembly through recruiting Cep152 and Plk4.

It may be hypothesized that the self-assembly of Deup1 via its N-terminus is sufficient for the spatial compartmentalization of centriole proteins. To address this possibility, we replaced the N-terminus region of human Deup1 (1–468 a.a.) with an optogenetic oligomerization tag (CRY2clust), which self-assembles into condensates under blue light (Park et al., 2017) (Fig. 6A). This system provided control over the assembly of the mScarlet I-tagged Deup1 C-terminus condensates in HeLa cells (Fig. 6B). As observed with the full-length Deup1, the CRY2clust fused-Deup1 C-terminus rapidly accumulated GFP-Cep152 at the condensates after photo-activation of CRY2clust; this effect was not observed with CRY2clust alone (Fig. 6C; Fig. S6A,D,E). We confirmed that the CRY2clust-Deup1 C-terminus did not concentrates GFP alone, indicating that the CRY2clust-Deup1 C-terminus specifically compartmentalizes GFP-Cep152 in the cytoplasm (Fig. S6C). Importantly, assemblies of the CRY2clust-Deup1 C-terminus accumulated GFP-Plk4, a master kinase for centriole biogenesis (Bettencourt-Dias et al., 2005; Habedanck et al., 2005), at the condensates when this protein was expressed along with Cep152-SNAP (Fig. 6D; Fig. S6F, G). These results demonstrate that self-assembly of Deup1 via its N-terminus drives the spatial compartmentalization of Cep152 and Plk4 in the cytoplasm. These data also suggest that the Deup1 N-terminus promotes its self-assembly to generate scaffolds in the cytoplasm, whereas the C-terminus directly binds to Cep152 to selectively compartmentalize procentriole components (Fig. 6E). From these findings, we propose that Deup1 self-assembly generates a structural core for deuterosomes, which dictates centriole biogenesis in multiciliogenesis through the accumulation of procentriole components (Fig. 6E).

## DISCUSSION

The deuterosome is a non-membranous organelle that supports *de novo* centriole biogenesis in multiciliogenesis. The mechanisms of deuterosome formation and the material properties of deuterosomes have been poorly understood. The findings of this study revealed that Deup1 assemblies, which presumably act as the core of deuterosomes, are stable structures in cells. Purified Deup1 protein self-assembles into stable and static condensates *in vitro*. Deup1 self-assembly relies on its N-terminus. Moreover, self-assembly of Deup1 drives the spatial compartmentalization of Cep152 and Plk4 in the cytoplasm. Based on these results, we propose that Deup1 self-assembly leads to the formation of the structural core of deuterosomes in an early stage of multiciliogenesis.

Recent studies have suggested that deuterosomes are assembled even in the absence of pre-existing centrioles (Mercey et al., 2019b; Nanjundappa et al., 2019; Zhao et al., 2019). In addition, it has been reported that Plk4 is not required for deuterosome assembly (Zhao et al., 2013). In line with these observations, we showed that Deup1 has the capacity to self-assemble into macromolecular structures even in the absence of centrioles and centriolar components. Given that Cep152 is implicated in the regulation of the number of deuterosomes per multiciliated cell (Zhao et al., 2013), the incorporation of Cep152 into Deup1 assemblies could promote the formation or stability of deuterosomes. A recent report demonstrated that Plk4 itself, unlike its kinase activity, is required for centriole formation in multiciliogenesis (Zhao et al., 2019). Therefore, we propose that the compartmentalization of Cep152-Plk4 induced by Deup1 self-assembly triggers centriole assembly around deuterosomes.

The present study shows that Deup1 forms static, stable assemblies in cells and *in vitro*. Recent studies have shown that the non-dynamic condensations of biomolecular components play various roles in cells (Boke et al., 2016; Laos et al., 2015; Schmidt and Görlich, 2015; Woodruff et al., 2017, 2018). It will be interesting to investigate whether the static condensation property of Deup1 assemblies is important for the function of Deup1 in the formation or maintenance of deuterosomes and in centriole biogenesis. We also speculate that static properties of Deup1 assemblies may be maintained during multiciliogenesis, because we observed that even at different stages of multiciliogenesis, turnover of exogenous Deup1 was similarly low (Fig. S2). However, we observed slight turnover of Deup1 in differentiating E1 cells, while less turnover was detected in undifferentiated E1 cells and *in vitro* (Figs 1F, 2B–D and 4A; Fig. S3B). This might be because of differences in the expression levels of Deup1, changes of cytoplasmic environments or interaction with other proteins.

Our results suggest that Deup1 self-assembles through multiple interactions between Deup1 proteins, because most fragments of Deup1 that we designed assembled into macromolecular structures in the cytoplasm (Fig. S5A– C). Deup1 contains multiple coiled coil domains, and these domains may mediate multiple interactions between Deup1 proteins. This was also observed in the self-assembly of SPD5 proteins, which are important for the formation of pericentriolar material (Woodruff et al., 2017).

We showed that the N-terminus region of Deup1 is required for the self-assembly, whereas the C-terminus recruits Cep152. In addition, we realized that the condensates formed with Deup1 N-terminus fragment slightly accumulated Cep152, although much less than those with Deup1 full-length (Fig. S6A–B). This result suggests that Deup1 N-terminus might act not only for self-assembly, but also for the recruitment of Cep152, although it remains unknown whether it is a direct or indirect interaction. Further investigations will be required to dissect the function of each Deup1 domain, for instance, by expressing Deup1 truncation mutants in differentiating E1 cells.

Deuterosomes exhibit spherical foci at the initial stage of multiciliogenesis, and subsequently increase in size and form ring-like structures at the later stage of multiciliogenesis in some multiciliated cells (Dirksen, 1971; Kim et al., 2018; Sorokin, 1968; Zhao et al., 2013, 2019). We hypothesize that molecular interactions between Deup1 assemblies and centriole components or procentriole assembly on the surface of Deup1 assemblies may influence the biophysical properties and shape of deuterosomes in the process of multiciliogenesis. It is also important to investigate the components of deuterosomes for a comprehensive understanding of deuterosome assembly. Further analyses, such as the proteomic analysis of deuterosomes, will provide new insights into the mechanisms underlying deuterosome formation.

## MATERIALS AND METHODS

### Cell culture

E1 cell line, a clonal cell line derived from a p53−/− mouse oviduct, was a gift from Dr Tadaaki Nakajima and Dr Yasuhiro Tomooka. Cells were maintained in DMEM/F12 without phenol red (nacalai tesque) supplemented with 10% FBS and 1% penicillin/streptomycin at 37°C in 5% CO_2_ atmosphere. For differentiation of E1 cells, cells were seeded at 8×10^4^ cells/insert onto culture insert (Millicell-PCF filter, 0.4 µm pore, Merck Millipore) coated with 0.05 mg/ml rat-tail collagen type I (Enzo). Before air liquid interface (ALI) culture, cells were cultured in the induction medium (DMEM/F12 without phenol red (nacalai tesque) supplemented with 10% Knockout serum replacement (Gibco), 10 µg/ml insulin (nacalai tesque), 10 µg/ml transferrin (nacalai tesque) and 1% penicillin/streptomycin) for 2 days. ALI was established by removing medium above the insert and by replacing medium at the basal side of the insert with the induction medium supplemented with 10 µM DAPT (MedChemExpress, HY-13027). Medium at the basal side of the insert was changed every 2–3 days. In order to induce protein expression under TRE promoter, 1 µg/ml Doxycycline was added to the culture medium from ALI 1.5 days.

HeLa cells were obtained from the ECACC and have been authenticated by STR profiling in ECACC. Cells were cultured in DMEM containing 10% FBS and 1% penicillin/streptomycin at 37°C in 5% CO_2_ atmosphere. It has been confirmed that cells were not contaminated with mycoplasma by indirect DNA staining using Hoechst 33258 with indicator cells (Vero cells).

### Lentivirus production and transduction

Lentivirus production was carried out by transfecting 293FT cells (ThermoFisher Scientific) with psPAX2 (Addgene#12260), pCMV-VSV-G (Addgene#8454) and the transfer plasmid using Lipofectamine 2000 (Life Technologies). Then, E1 cells were infected with the lentivirus. Transformed E1 cells were selected with medium containing 10 µg/ml Blasticidin or 2 µg/ml Puromycin for 4 days.

### cDNA cloning

cDNAs of Deup1 (NP_001360846) and Centrin2 (NP_062278.2) were cloned from mouse lung cDNA library using following primers: (Deup1, 5′-ATGGAGAACCAAGCCCATACCACAGCAG-3′ and 5′-TCATATGTGTCTACTCTGCTTGAGTTTGG-3′) and (Centrin2, 5′-TAGCGTGCCACCATGGCCTCTAATTTTAAGAAGACAAC-3′ and 5′-TGATCCAGAACCATAGAGGCTGGTCTTTTTCATGATGCG-3′). cDNA of a short isoform of mouse Deup1 (NP_001360849.1) was made through PCR mutagenesis. To prepare cDNA of human Deup1 (NP_857596.2), its partial cDNA (corresponds to 264-604 a.a. region) was obtained from Sino Biological (#HG14376-G), while the other region (corresponds to 1-263 a.a. region) of human Deup1 was synthetically generated from mouse Deup1 cDNA by PCR mutagenesis to become the amino acid sequence of human Deup1. Unless otherwise noted, a longer isoform of mouse Deup1 (NP_001360846) was analyzed in this study.

### Plasmids

To generate E1 cells expressing mScarlet I-Deup1 and Centrin2-GFP, cDNA encoding Cas9 in pCW-Cas9 (Addgene#50661) was replaced with cDNA encoding mScarlet I-Deup1 or Centrin2-GFP. To select transduced cells with a blastcidin resistant gene, cDNA encoding a puromycin resistant gene in pCW-Centrin2-GFP was replaced with cDNA encoding a blastcidin resistant gene. For ectopic protein expression of Deup1 in mammalian cells, cDNA encoding Deup1 was cloned into pcDNA5/frt/to (ThermoFisher Scientific), pCMV-3FLAG and pEGFPC1. GFP-Plk4 was expressed using pcDNA5/frt/to. Ectopic expression of GFP-Cep152 and Cep152-SNAP in HeLa cells was driven by CMV promoter using pEGFPC1. For protein expression in *E.coli*, cDNAs encoding mScarlet I, mScarlet I-Deup1 and SNAP-Halo were cloned into pGEX 6p-1 (GE healthcare) and modified by inserting 6×His-tag cDNA. Thus, GST-tag and His-tag were fused to the N-terminus and the C-terminus of the recombinant protein, respectively. For the cleavage of His-tag, cDNA sequence encoding a TEV protease recognition site was inserted. For protein expression of GST-Cep152-SNAP-Halo-His_6_ in Sf9 cells, pLIB (Addgene#80601) was used. Optogenetic analyses were carried out using mCherry-CRY2clust (Addgene#105624). Plasmid construction was performed using PrimeSTAR mutagenesis basal kit (Takara) and In-Fusion Cloning kit (Takara). Deup1 mutants were made through PCR mutagenesis.

### Antibodies

The following primary antibodies were used: rabbit polyclonal antibodies against GFP (MBL, 598, IF 1:1000), RFP (MBL, PM005, IF 1:1000), Deup1 (Proteintech, 24579-1-AP, IF 1:50), Myc (Santa Cruz Biotech, sc-789, IF 1:1000), γ-Tubulin (Sigma-Aldrich, T5192, IF 1:500); mouse monoclonal antibodies against Centrin-2 (Merck Millipore, clone 20H5, 04-1624, IF 1:1000), FoxJ1 (eBioscience, 2A5, IF 1:500), GFP (Invitrogen, A11120, IF 1:1000), FLAG (Sigma-Aldrich, F1804, IF 1:1000), RFP (MBL, M208-3, IF 1:1000), acetylated tubulin (Sigma-Aldrich, T7451, IF 1:1000): rat monoclonal antibodies against ZO-1 (Santa Cruz, sc-33725, IF 1:1000). The following secondary antibodies were used: Alexa Fluor 488 goat anti-mouse IgG (H+L) (Molecular probes, A11001, IF 1:1000), Alexa Fluor 488 goat anti-rabbit IgG (H+L) (Molecular probes, A11008, IF 1:1000), Alexa Fluor 594 goat anti-mouse IgG (H+L) (Molecular probes, A11005, IF 1:1000), Alexa Fluor 568 goat anti-rabbit IgG (H+L) (Molecular probes, A11011, IF 1:1000), Cy5 goat anti-rat IgG (H+I) (Thermo Fisher Scientific, A10525, IF 1:1000).

### Plasmid transfection into mammalian culture cells

Transfection of plasmid DNA was performed using Lipofectamine 2000 (Life Technologies), respectively, according to the manufacturer's instructions. Transfected cells were analyzed 18–24 h after transfection with plasmid DNA.

### Protein purification

*E.coli* strain BL21 gold (DE3) was used for Deup1 protein expression. Protein expression was induced at 18°C for 16 h by incubating in LB medium supplemented with 0.3 mM IPTG. Cell pellets were suspended in lysis buffer [50 mM Tris (pH7.5), 500 mM NaCl, 5% Glycerol, 20 mM Imidazole, 0.1% CHAPS, 1 mM β-ME, 1 mM PMSF] and lysed by lysozyme treatment and sonication. The lysates were then centrifuged at 9000× ***g*** for 45 min and the supernatants were collected. The supernatants were incubated with Ni-NTA Agarose beads (Qiagen) at 4°C for 1 h. The beads were washed with first wash buffer (lysis buffer supplemented with 40 mM Imidazol). Elution was performed in first elution buffer (lysis buffer supplemented with 300 mM Imidazole). The eluates were incubated with glutathione sepharose beads (GE healthcare) at 4°C for 1 h. The beads were washed with second wash buffer (lysis buffer without Imidazole) and then washed with pre-elution buffer [50 mM Tris (pH7.5), 500 mM NaCl, 5% Glycerol, 1 mM β-ME]. Elution was performed in second elution buffer [pre-elution buffer supplemented with GST-tagged PreScission protease and His-tagged TEV protease (GenScript)] at 4°C, overnight. His-tagged TEV protease was removed by adding Ni-Agarose beads. The supernatant was collected and used. Protein concentration was determined by Bradford assay. For protein purification from Sf9 cells, cells were maintained in Sf-900 II SFM (ThermoFisher Scientific). Protein expression was induced by baculovirus mediated system and the cells were harvested 72 h post infection. Proteins were purified as described above.

### Imaging of purified proteins

Fluorescence-labeled proteins were mixed with buffer solution containing PEG [Final concentration: 50 mM Tris (pH7.5), 150 mM NaCl, 1.5% Glycerol, PEG8000 (Promega, V3011) with the indicated concentration] and incubated at RT for 10 min. To examine the effect of bovine serum albumin (BSA), BSA was dissolved in a buffer solution containing 50 mM Tris (pH7.5) and 150 mM NaCl and tested as a molecular crowding instead of PEG. SNAP labeling of purified proteins was performed by incubating 30 nM of purified proteins with 300 nM of SNAP-Cell 647-siR (NEB, 591025) and 1 mM DTT at RT for 2 h. The samples were mounted onto slide glasses (Matsunami, S0318) and covered with cover glasses (Matsunami, C015001). Images were taken using Leica TCS SP8 inverted confocal microscope equipped with a Leica HCX PL APO×63/1.4 oil CS2 objectives and excitation wavelength 552 and 638 nm. The fields were chosen randomly. The experiments were repeated at least twice. For counting *in vitro* condensates per field (18.49×18.49 µm), fluorescence signals above the defined threshold intensity and size were regarded as condensates and the numbers were measured using Particle analysis in Fiji (NIH). The diameter of condensates was measured using Fiji.

### Immunofluorescence

For immunostaining, cells were seeded on coverslips (Matsunami, C015001) or culture inserts and fixed with cold Methanol at -20°C for 7 min. Cells were washed with PBS for 5 min three times and incubated in blocking buffer (1% BSA, 0.05% Triton X-100 in PBS) for 30 min. Cells were then incubated with primary antibodies in blocking buffer at room temperature for 1 h or at 4°C overnight and washed with PBS twice or three times, and incubated with secondary antibodies for 1 h at RT. DNA was stained with Hoechst 33258 (DOJINDO) during or after incubation with secondary antibodies. Cells were washed with PBS twice or three times and then mounted onto slide glasses.

For imaging of fixed cells, Zeiss Axio Imager M2 equipped with a 63×/NA 1.4 Plan-APOCHROMAT oil objective and an AxioCam HRm camera was used. The images were collected at 0.25 µm z-steps. Leica TCS SP8 inverted confocal microscope equipped with a Leica HCX PL APO 63×/NA 1.4 oil CS2 objective and excitation wave length 405, 488, 552 and 638 nm was also used. The fields were chosen randomly. The experiments were repeated at least twice. Deconvolution was performed using Huygens essential software (SVI). Z-projection was performed using maximum intensity projection in Fiji. In Fig. S1A, cells on the edge of images were excluded from the measurement because of the difficulty to distinguish whether they have multicilia or not.

### Fluorescence recovery after photobleach (FRAP)

For FRAP analysis of undifferentiated cells, cells were cultured in medium containing HEPES on 35 mm glass-bottom dishes (Greiner-bio-one, #627870). For analysis of differentiating E1 cells (ALI for 5 days), the membranes were cut out from the culture insert. Then, the membranes were inverted onto a glass-bottom dish. The membranes were incubated with the induction medium supplemented with 1 µg/ml Doxycyclin.

FRAP analysis was performed using Leica TCS SP8 inverted confocal microscope equipped with a Leica HCX PL APO×63/1.4 oil CS2 objectives in a chamber with or without 5% CO_2_ at 37°C. The pinhole was adjusted at 2.0 airy units. Single section images were recorded at 1.29 s (pre-bleach) and 5 s (for undifferentiated E1 cells) or 10 s (for differentiating E1 cells and purified proteins) (post-bleach) intervals. A region of interest (encircled with 1.2 µm diameter for E1 cells or 0.7 µm diameter for purified proteins) around the mScarlet I or GFP signals was bleached with maximum laser power. Mean intensity values of the mScarlet I or GFP signals [encircled with 1.01 µm (for differentiating E1), 0.95 µm (for undifferentiated E1) and 0.65 µm (for purified proteins) diameter] were measured using Fiji (NIH) and cytoplasmic signals for cells or signals outside of the condensates for purified proteins were subtracted as background. Intensities at unbleached condensates were also measured and shown in each graph. Signal intensity was normalized with the average of three pre-bleach signals.

### Optogenetics

HeLa cells were seeded onto 35 mm glass-bottomed dishes (Greiner-bio-one, #627870). Leica TCS SP8 inverted confocal microscope equipped with a Leica HCX PL APO×63/1.4 oil CS2 objectives and excitation wave length 488 and 552 was used. Cells were maintained in a chamber in 5% CO_2_ at 37°C during the experiments. Condensation of CRY2clust was induced with blue light (488 nm). The images were collected every 4 s. Partition coefficients were defined as the ratio of concentrations of GFP-tagged protein in condensates versus the cytoplasm and calculated by dividing the mean fluorescence intensity inside the CRY2clust condensates by the mean fluorescence intensity of the cytoplasm.

### Protein sequence analysis

Coiled coil regions and intrinsically disordered regions were predicted using coiled coil prediction tool from PRABI-Lyon-Gerland and PrDOS, respectively.

### Statistics

Statistical analyses were performed using R statistical software. Prior to statistical analysis, data were subjected to Shapiro–Wilk normality test. Statistical test, sample sizes and *P* values are described in each figure legend.

## Supplementary Material

Supplementary information
