# Supplementary material for: Biophysical and biochemical properties of Deup1 self-assemblies: a potential driver for deuterosome formation during multiciliogenesis
Source: Biol Open. 2021 Mar 3;10(3):bio056432. doi: 10.1242/bio.056432 (PMC7938805; doi:10.1242/bio.056432)
Supplement: Supplementary information [file biolopen-10-056432-s1.pdf]

Supplementary information

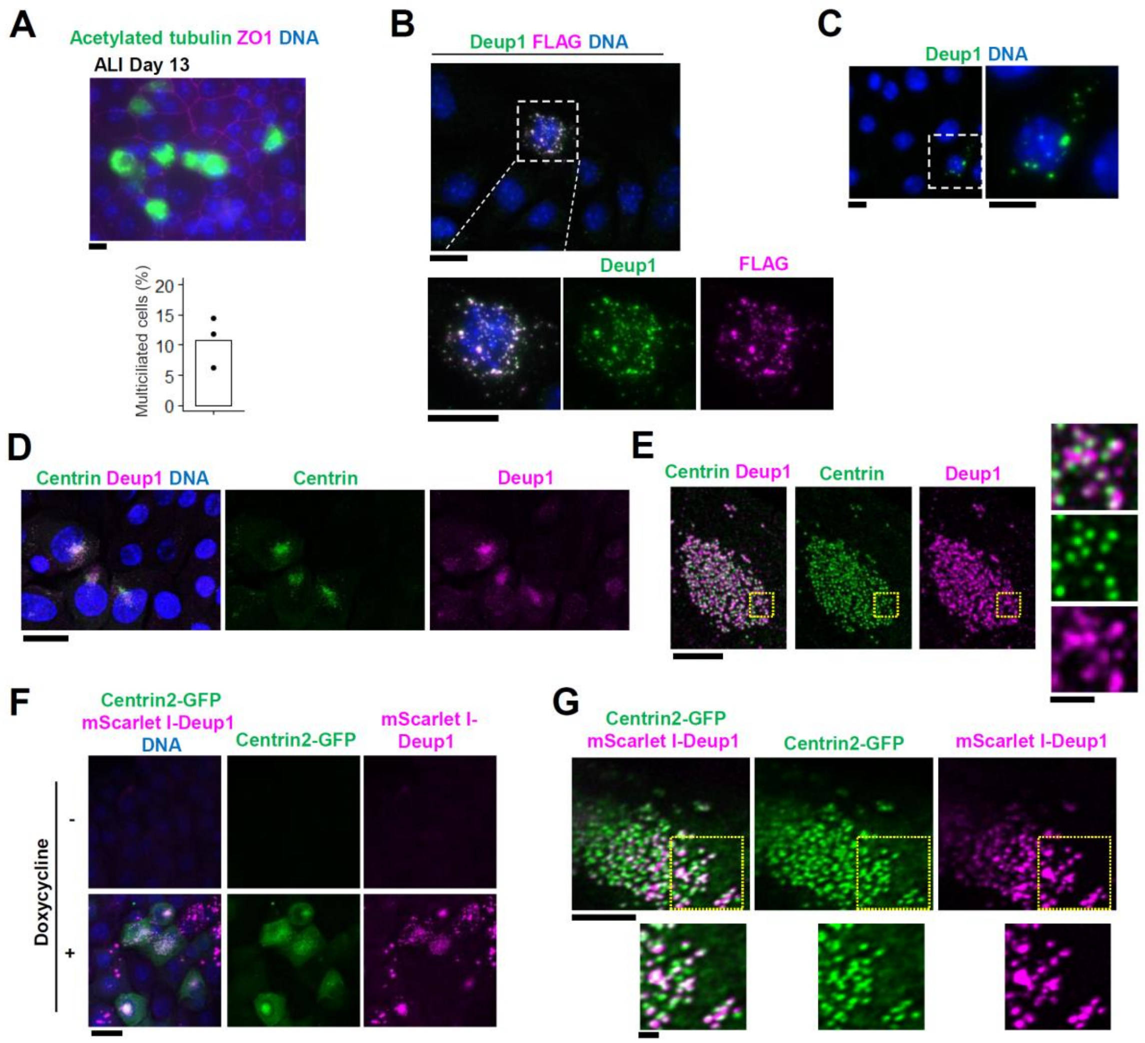

Fig. S1. Characterization of E1 cells, related to Fig.1

(A) Differentiation efficiency of E1 cells. E1 cells were cultured in ALI (air-liquid interface) for 13 days and fixed for immunostaining. Green, magenta and blue represent acetylated tubulin, ZO1 and DNA, respectively. Graph shows mean percentage of multiciliated cells from three independent experiments (309, 576 and 326 cells were measured, respectively). Scale bar, 10 µm. **(B)** Validation of specificity of anti-Deup1 antibodies. Undifferentiated E1 cells expressing Deup1-3xFLAG were immunostained with anti-Deup1 and anti-FLAG antibodies. Scale bar, 20 µm. **(C)** Undifferentiated cells expressing Deup1 (without any tag). Deup1 was stained with anti-Deup1 antibodies. Scale bar, 10 µm. **(D)** and **(E)** Immunostaining of differentiated E1 cells (ALI 4 days) with anti-Deup1 and anti-Centrin antibodies. (D) Scale bar, 20 µm. (E) Scale bar, 5 µm; magnified image, 1 µm. (E) Deconvoluted images were shown. The diameter of Deup1 assemblies in the E1 cell shown in (E) was 337 ± 224 nm (Mean ± SD). **(F)** and **(G)** Doxycycline-dependent expression of Centrin2-GFP and mScarlet I-Deup1 in E1 cells. Immnostaining of mScarlet I-Deup1 and Centrin2-GFP in

Biology Open • Supplementary information

differentiating E1 cells (ALI 5 days). (F) Scale bar, 20 μm. (G) Deconvoluted images were shown. Scale bar, 5 μm; magnified image, 1 μm.

Biology Open • Supplementary information

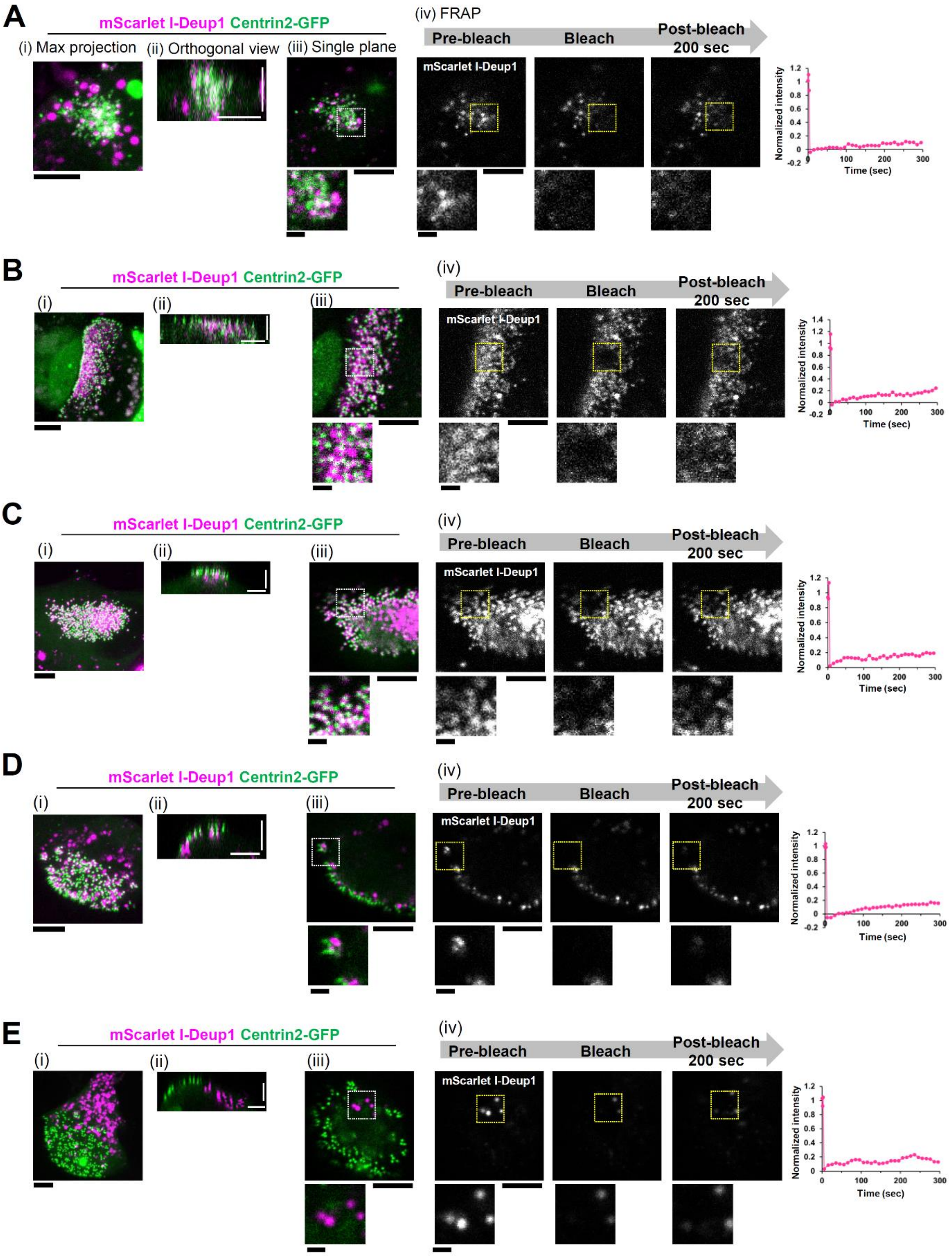

Fig. S2. FRAP analysis of mScarlet I-Deup1 in differentiating E1 cells, related to Fig.1F

(A)-(E) Representative images and quantification of FRAP analysis of mScarlet I-Deup1 in differentiating E1 cells (ALI 5 days). (i) max intensity projection, (ii) an orthogonal view (single plane), (iii) single plane just before FRAP experiment, (iv) during FRAP analysis (single plane). Graphs show individual data of

Biology Open • Supplementary information

FRAP analysis. Intensities were normalized with the average of three pre-bleach signals. Scale bar, 5 µm; magnified image, 1 µm. **(A)** Centrioles are dispersed in Z-axis. **(B)** Some centrioles are dispersed in Z-axis and some centrioles show apical alignment. **(C)-(D)** Centrioles are aligned at the apical side of cells. (D) is also presented in Fig.1F. **(E)** Deup1 foci are not associated with centrin foci. We assume that this cell is probably at the late stage of centriole amplification showing centriole-deuterosome dissociation. This data was not included in Fig.1F in order to focus on the properties of Deup1 foci associated with centrioles.

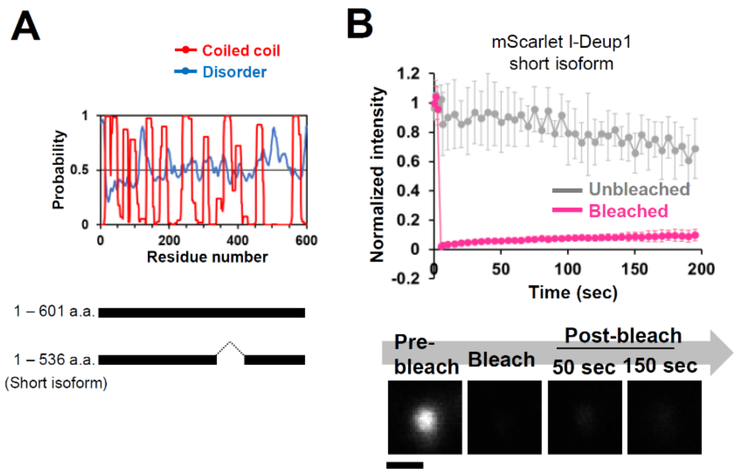

Fig. S3. Characterization of Deup1 short isoform

(A) Comparison of two Deup1 isoforms. The shorter isoform (Full length 536 a.a.) lacks 347-411 a.a. region of the longer isoform (Full length 601 a.a.). **(B)** FRAP analysis of mScarlet I-Deup1(shorter isoform) in undifferentiated E1 cells. Scale bar, 10 µm; magnified image, 1 µm. Intensities were normalized with the average of three pre-bleach signals. Graph shows mean ± SD of 7 cells from two independent experiments.

Biology Open • Supplementary information

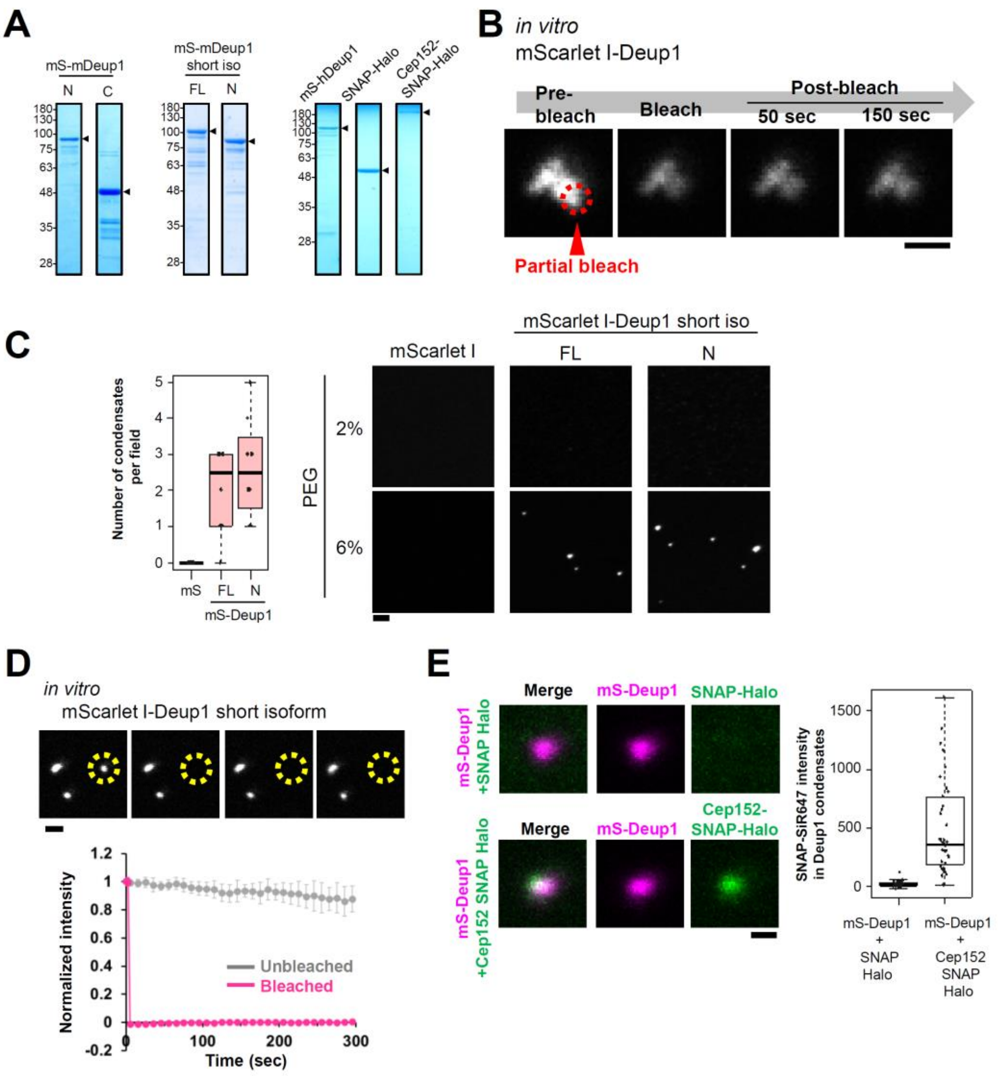

Fig. S4. Characterization of Deup1 *in vitro* and in cells

(A) CBB staining of purified proteins used in this study. Arrowheads refer to the purified target proteins. **(B)** Partial bleaching of mScarlet I-Deup1. Purified mScarlet I-Deup1 was incubated in a buffer solution containing 6% PEG for 90 min. Scale bar, 1 µm. **(C)** Condensation of mSarllet I-Deup1 shorter isoform *in vitro*. Graphs represent quantification for 6%PEG condition and show box (25 to 75%), whisker (10 to 90%). Lines in graphs indicates medians. n = 12 fields per condition. Scale bar, 2 µm. **(D)** FRAP analysis of mScarlet I-Deup1 short isoform. Intensities were normalized with the average of three pre-bleach signals. Graph shows mean ± SD of 6 condensates from two independent experiments. Scale bar, 1 µm. **(E)** mScarlet I-Deup1 (human) proteins were mixed with SNAP-Halo or Human Cep152-SNAP-Halo. SNAP-tagged proteins were labeled with SNAP-647-SiR dye. Scale bar, 0.5 µm. Graph indicates SNAP-647-SiR intensity in Deup1 condensates after subtraction of background intensity. n = 32 (SNAP-Halo) and 41 (Cep152-SNAP-Halo) condensates.

Biology Open • Supplementary information

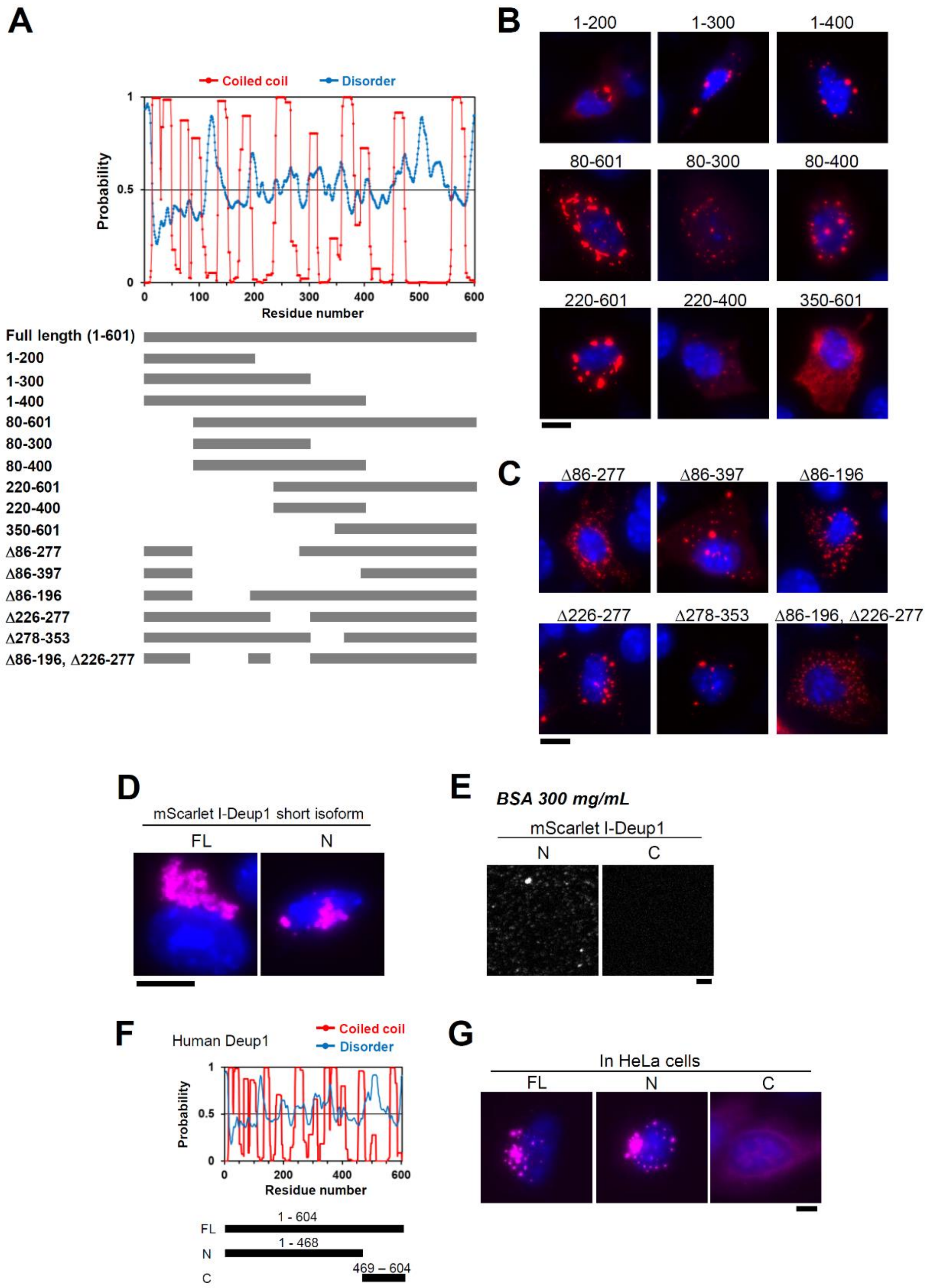

Fig. S5. Characterization of Deup1 *in vitro* and in cells

(A) Prediction of coiled coil and disordered regions in Deup1 structure. Regions of Deup1 fragments are shown. **(B)** and **(C)** Ectopic expression of mScarlet I-Deup1 fragments in undifferentiated E1 cells. Scale bar, 10 µm. **(D)** Ectopic expression of mScarlet I-Deup1 short isoform in undifferentiated E1 cells. N-terminus (N), 1-401 a.a.. Scale bar, 10 µm. **(E)** Effects of BSA (300 mg/mL) on mScarlet I-Deup1 fragments *in vitro*. Scale bar, 2 µm. (**F**) Prediction of coiled coil regions and disordered regions in human Deup1. **(G)**

Biology Open • Supplementary information

mScarlet I-human Deup1 fragments were ectopically expressed in HeLa cells. Full length, 1-604 a.a.. N-terminus, 1-468 a.a.. C-terminus, 469-604 a.a. Scale bar, 5 μm.

Biology Open • Supplementary information

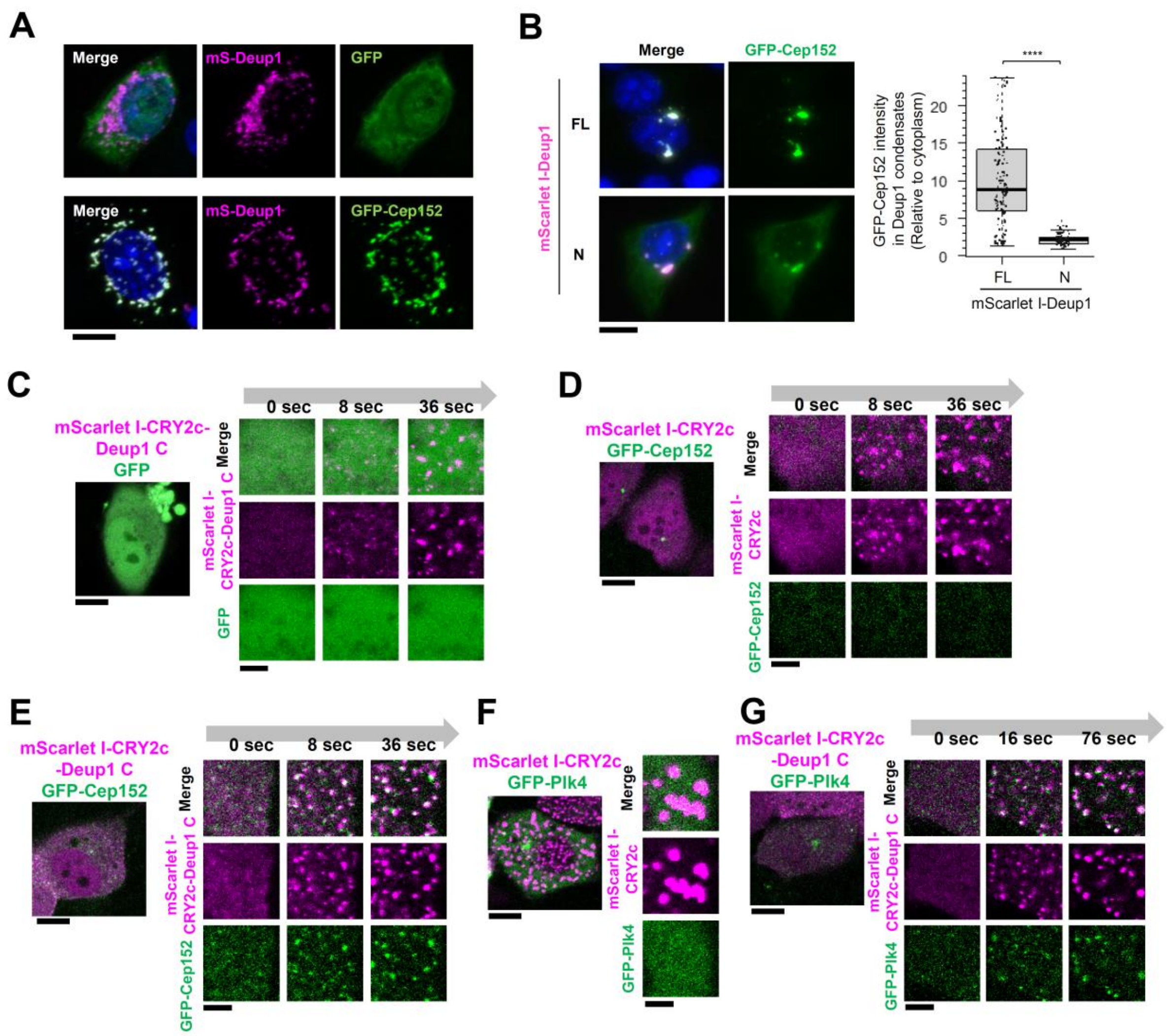

Fig. S6. Deup1 assemblies specifically accumulate Cep152 and Plk4

(A) Ectopic expression of mScarlet I- Human Deup1 and GFP or GFP-Cep152 in HeLa cells. Scale bar, 10 μm. **(B)** Co-expression of GFP-Cep152 with mScarlet I-Human Deup1 full length or N-terminal fragment (1-468 a.a.) in HeLa cells. GFP-Cep152 intensities in Deup1 condensates relative to the cytoplasmic intensities were measured. Graphs show box (25 to 75%), whisker (10 to 90%). Lines in graphs indicate medians. n = 138 and 82 condensates (from 40 and 36 cells, respectively). The sample size was determined based on the reproducibility of the data. ****, $p < 0.0001$ (Mann-Whitney U test). Scale bar, 10 μm. **(C)** Co-expression of GFP with mScarlet I-CRY2clust-Deup1-C in HeLa cells. The representative cell is same as shown in Fig. 6B. **(D)** Co-expression of GFP-Cep152 with mScarlet I-CRY2clust in HeLa cells. **(E)** Co-expression of GFP-Cep152 with mScarlet I-CRY2clust-Deup1-C in HeLa cells. **(F)** Co-expression of GFP-Plk4 with mScarlet I-CRY2clust in HeLa cells. Cep152-SNAP was co-expressed. **(G)** Co-expression of GFP-Plk4 with mScarlet I-CRY2clust-Deup1-C in HeLa cells. Cep152-SNAP was co-expressed. Scale bar, 10 μm; magnified image, 2 μm. Time after the induction with blue light is shown. Non-magnified images show 0 sec after the blue light induction.
